# Editorial: Based models and machine learning on CT, MRI and PET-CT in head and neck cancer diagnosis, staging and outcome prediction

**DOI:** 10.3389/fradi.2026.1963901

**Published:** 2026-08-25

**Authors:** Emma Gangemi, Marco Panfili, Antonello Vidiri

**Affiliations:** 1 Radiology and Diagnostic Imaging, IRCCS National Cancer Institute Regina Elena, Rome, Italy; 2 Department of Neuroradiology, Fondazione Policlinico Universitario Agostino Gemelli IRCCS, Rome, Italy

**Keywords:** CT, head and neck tumors, machine learning, MRI, PET-CT

Head and neck squamous cell carcinoma (HNSCC) represents the seventh most prevalent cancer worldwide, with approximately 940,000 new cases and 480,000 deaths annually (1). The incidence of oropharyngeal cancer is rising globally, largely driven by human papillomavirus-related infections (HPV). In HNSCC post-treatment recurrences and metastases remain common occurrences that contribute to poor prognosis, because this disease is characterized by marked heterogeneity in anatomical presentation, tumor biology, and treatment outcomes, posing substantial challenges in selecting optimal management strategies. While multi-modal treatments —including surgery, radiotherapy, chemotherapy, and immunotherapy— remain the cornerstone of management, the integration of artificial intelligence (AI) into clinical workflows could offer transformative potential for precision oncology (2). The convergence of large imaging datasets, enhanced computational power, and refined machine learning (ML) and deep learning (DL) algorithms has advanced AI applications across the head and neck cancer (HNC) continuum. Recent systematic reviews have demonstrated that ML models consistently outperform traditional linear approaches in predicting post-treatment survival and disease progression, with concordance indices ranging from 0.70 to 0.79 (3). Similarly, radiomic analysis of multi-modal imaging has emerged as a powerful tool for non-invasive tumor characterization (4, 5), enabling automated segmentation, treatment response prediction, and prognostic stratification (6, 7).

This Research Topic brings together eight contributions spanning diagnostic imaging, predictive modeling, and therapeutic assessment

In the diagnostic realm, ML improves the accuracy of differential diagnosis for complex head and neck lesions. Qin et al. performed a systematic review and meta-analysis of 17 studies involving 3,321 participants for identifying sinonasal inverted papilloma, demonstrating that radiomics-clinical fusion achieves superior diagnostic performance (sensitivity 0.85, specificity 0.87) compared to imaging alone. Yuan et al. developed a random forest model integrating contrast-enhanced ultrasound and clinical data to predict lymph node metastasis in clinically node-negative papillary thyroid carcinoma, with AUCs exceeding 0.92. This non-invasive approach offers a practical tool to optimize surgical extent and reduce unnecessary prophylactic dissections.

AI also advances staging precision by predicting metastatic dissemination beyond locoregional disease. Zhang et al., 2026 developed a multimodal DL framework integrating 18F-FDG PET/CT imaging, radiomic features, and clinical parameters to predict synchronous distant metastasis in nasopharyngeal carcinoma. Dual-modal networks outperformed single-modality approaches, with ResNet18 achieving an internal ROC-AUC of 0.804. The optimal Multilayer Perceptron (MLP) hybrid model reached an internal ROC-AUC of 0.839 and was externally validated with an ROC-AUC of 0.701. Notably, conventional T and N staging alone demonstrated poor discriminative capacity, whereas DL integration substantially improved predictive performance, enabling early identification of high-risk patients for tailored therapeutic strategies.

AI also plays a pivotal role in perioperative clinical management. Zhang and Zhou presented a case report on multimodal imaging-guided awake transnasal fiberoptic intubation in an 80-year-old patient with a giant palatal pleomorphic adenoma. Through integration of MRI, 3D CT reconstruction, and fiberoptic laryngoscopy, the team delineated tumor-airway relationships, identified a safe nasal corridor, and successfully performed awake intubation—avoiding tracheotomy. This exemplifies how AI-enhanced anatomical mapping can inform personalized airway strategies while ensuring patient safety in high-risk scenarios.

A critical area where AI is reshaping practice is automated image analysis. Accurate delineation of gross tumor volumes (GTV) and organs at risk remains fundamental to radiotherapy planning, yet manual contouring is labor-intensive and subject to significant inter-observer variability. Schanne et al. evaluated a DL-based PET/CT segmentation model under clinically relevant imaging perturbations. While 3D Dynamic U-Net architectures achieved promising baseline performance with Dice scores of 0.766 for primary tumors, nodal segmentation remained vulnerable to spike noise and bias artifacts, with clinical usability declining sharply under severe perturbations. This underscores the need for targeted data augmentation and real-world validation before clinical deployment.

The application of AI extends to treatment-specific outcome prediction. Jungbauer et al. demonstrated that automated CT-based skeletal muscle-to-bone ratio provides independent prognostic information for HNSCC patients receiving immune checkpoint inhibitors (HR: 0.25, 95% CI: 0.1–0.64). Combined with serum albumin, this enabled refined risk stratification with markedly divergent survival outcomes. This is particularly relevant given the expanding role of immunotherapy in recurrent and metastatic HNSCC, where biomarkers of treatment tolerance and response remain urgently needed.

Moreover, AI unlocks prognostic insights from routine imaging. Pei et al. developed a dual-energy CT (DECT)-based nomogram integrating normalized iodine concentration with neutrophil-to-lymphocyte ratio and lactate dehydrogenase to predict progression-free survival in nasopharyngeal carcinoma, achieving a C-index of 0.88. Jian et al. conducted the first network meta-analysis comparing MRI and CT radiomics for chemotherapy response prediction, favoring MRI-based nonlinear algorithms (support vector machine-SVM) and clinical-radiomics fusion. These studies demonstrate radiomics transitioning toward clinically actionable tools, though IBSI/METRICS standardization and prospective multicenter validation remain essential.

Together, these contributions reveal a field in rapid evolution, yet several challenges persist. Standardization of image acquisition protocols, feature extraction pipelines, and model validation frameworks remains essential to ensure reproducibility and generalizability. The integration of multi-modal imaging data requires robust harmonization to address vendor-specific variability. Furthermore, clinical translation necessitates prospective multicenter validation, regulatory frameworks, clinician education, and seamless integration into existing electronic health record systems.

As AI continues to mature, its role in HNC will likely expand from assisting decision-making to enabling fully personalized treatment pathways that dynamically adapt to individual patient profiles. The contributions assembled in this Research Topic demonstrate that AI is no longer a distant prospect but an emerging reality with tangible applications across the care continuum. By leveraging these collective advances, the head and neck oncology community is well-positioned to improve patient outcomes through precise diagnosis, tailored therapy selection, and dynamic prognostic monitoring—ultimately realizing the promise of personalized medicine in this challenging disease.
